# Strain selection and adaptation of a fungal-yeast-microalgae consortium for sustainable bioethanol production and wastewater treatment from livestock wastewater

**DOI:** 10.1186/s12934-024-02537-4

**Published:** 2024-10-22

**Authors:** Salma B. Abdalla, Reda M. Moghazy, Ahmed A. Hamed, Mohamed O. Abdel-Monem, Mohamad A. El-Khateeb, Mervat G. Hassan

**Affiliations:** 1 Water Pollution Research Department, National Research Centre, 33 El-Buhouth Street, P.O. Box 12622, Dokki, Giza Egypt; 2 Microbial Chemistry Department, National Research Centre, 33 El-Buhouth Street, P.O. Box 12622, Dokki, Giza Egypt; 3 Faculty of Science, Botany and Microbiology Department, Benha University, Benha, Egypt

**Keywords:** Fungi, Yeast, Microalgae, Livestock wastewater, Bioethanol production

## Abstract

This study explores the potential of strain selection and adaptation for developing a fungi-yeast-microalgae consortium capable of integrated bioethanol production and livestock wastewater treatment. We employed a multi-stage approach involving isolation and strain selection/adaptation of these consortiums. The study started with screening some isolated fungi to grow on the cellulosic biomass of the livestock wastewater (saccharification) followed by a fermentation process using yeast for bioethanol production. The results revealed that *Penicillium chrysogenum (Cla)* and *Saccharomyces cerevisiae (Sc)* produced a remarkable 99.32 ppm of bioethanol and a concentration of glucose measuring 0.56 mg ml^− 1^. Following the impact of fungi and yeast, we diluted the livestock wastewater using distilled water and subsequently inoculated Nile River microalgae into the wastewater. The findings demonstrated that *Chlorella vulgaris* emerged as the dominant species in the microalgal community. Particularly, the growth rate reached its peak at a 5% organic load (0.105385), indicating that this concentration provided the most favorable conditions for the flourishing of microalgae. The results demonstrated the effectiveness of the microalgal treatment in removing the remaining nutrients and organic load, achieving a 92.5% reduction in ammonia, a 94.1% reduction in nitrate, and complete removal of phosphate (100%). The algal treatment also showed remarkable reductions in COD (96.5%) and BOD (96.1%). These findings underscore the potential of fungi, yeast, and Nile River microalgae in the growth and impact on livestock wastewater, with the additional benefit of bioethanol production.

**Graphical abstract:**

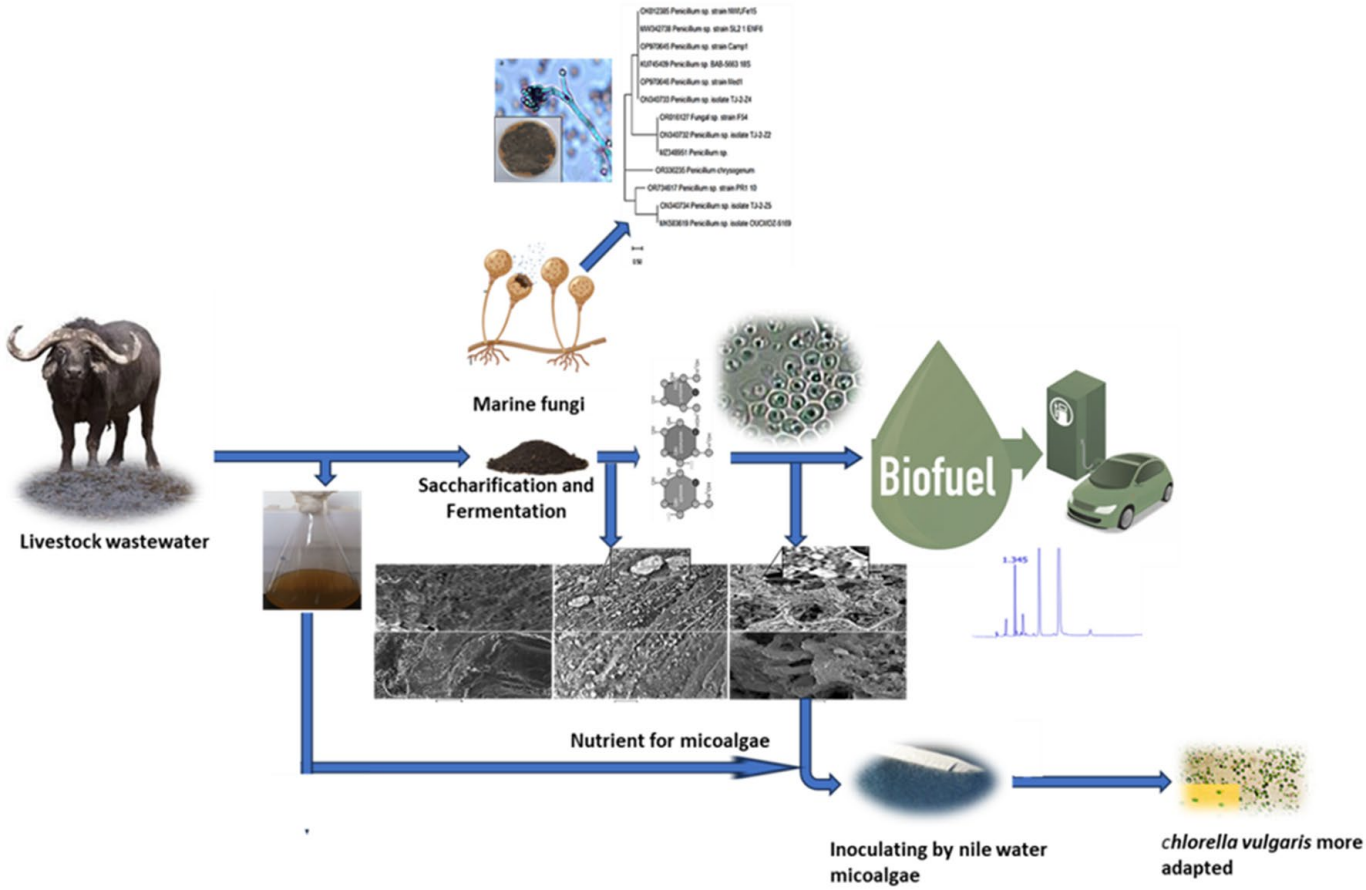

## Introduction

The world population is growing, and industrialization is accelerating, which has caused substantial alterations in the dietary structure of the public. For instance, The amount of meat consumed per person has increased by 50% over the past 30 years, whereas, there has been a double increase in the demand for dairy products such as milk and cheese and eggs [1, 2]. Livestock wastewater (LW) contains major organic pollutants such as carbohydrates, proteins, and lipids from milking. (LW) also has a high concentration of color, suspended solids, COD, BOD, nutrients, antibiotics, pathogenic bacteria [3], yeasts, molds, etc., that, if not handled and treated appropriately, turn into toxic ones that should not be discharged on farms and into water bodies [2, 4]. Additionally, The pollutants also represent a risk to human health and slowly degrade the quality of the air, groundwater, and soil. For this reason, it is crucial to manage these large amounts of LW in an economical, sustainable, and ecologically friendly manner [2].

Conventional wastewater treatment often relies on physical, chemical, and biological processes, which can be costly, inefficient, and generate significant sludge. Our proposed integrated approach, combining yeast fermentation and microalgal bioremediation, offers a more sustainable and effective solution by enhancing pollutant removal, recovering valuable resources, and reducing environmental impact. This innovative method aims to address the limitations of traditional wastewater treatment while providing a circular approach to resource management [1, 5, 6].

A combined culture of yeasts and microalgae is used as an effective, integrated, environmentally friendly method for treating wastewater from a distillery and wastewater treatment plant [7]. Yeasts might use microalgae to produce oxygen, and microalgae could use yeasts to produce carbon dioxide. The primary function of yeasts is to absorb organic matter, and microalgae need nutrients from wastewater. This resulted in a notable improvement in the generation of lipids and the elimination of organic materials and nutrients from animal effluent [8–11]. Microalgae and certain microorganisms like yeast and bacteria integration were recently considered as promising alternative ways to traditional oil crops for lipid production and biodiesel generation. While these organisms can accumulate high lipid content, problems such as low productivity and the production of undesirable lipid types hinder their commercialization. To overcome these limitations, strategies like co-cultivation, metabolic engineering, and optimized growth conditions are being explored to enhance lipid yield and quality. A deeper understanding of lipid biosynthesis pathways in these microorganisms is crucial for developing efficient and economically viable biofuel production processes [12–15]. Because of their many functions, fungi, a well-represented member of the wastewater microbial population, can be shown to be extremely beneficial and exploitable organisms [16]. Fungi are incredibly adaptable to harsh environments and fast-changing conditions. For example, they may readily adapt to many kinds of municipal wastewater [17–19].

Because of fungal ability to produce extracellular enzymes such as laccase and peroxidase that break down complicated and possibly dangerous compounds such as pollutants including synthetic dyes, chlorophenols, polychlorinated biphenyls, and polycyclic aromatic hydrocarbons [20–28]. Biological yeasts are eukaryotic, multicellular microorganisms that belong to the kingdom of fungi. There are many kinds of yeast strains on the market anywhere in the world. Yeast is typically employed in conventional fermentation procedures. Different strains of Saccharomyces were employed in the procedures of yeast fermentation. According to scientists, S. cerevisiae was the most productive form of yeast in a variety of tests out of all the varieties [29–32]. The Japanese Research Institute implemented yeast wastewater treatment technology for the first time globally in the early 1990s. with advancements in research, In recent years, new technologies centered around yeast have become increasingly prevalent in the water treatment industry [33]. Since yeast wastewater treatment technologies have advanced, it has been discovered that yeast produces glycolipids, lipids, and enzymes [34, 35]. Because of this, it is frequently employed in the treatment of highly concentrated and very valuable organic wastes [8].

The most common, traditional, and well-researched natural metabolic pathway for turning lignocellulosic biomass into the most significant alcohol, bioethanol (C2H5OH), is the fermentation method. Through the evolution of carbon dioxide (CO_2_), an organism changes complex carbohydrates into simple sugar and sugar into an acid or alcohol in this process [32, 36, 37]. According to the Emden-Meyerhoff pathway (EMP), the process is an anaerobic fermentation that is aided by enzymes generated by fungus and bacteria. Yeast, bacteria, or enzymes are used in this fermentation process in experiments [36]. Another method that was developed more recently depends on using certain kinds of biomass to eliminate contaminants. Microalgae have the most intriguing and widely utilized type of substitute biomass in wastewater treatment applications nowadays [38, 39], They are a varied class of unicellular photosynthetic organisms that can develop and even flourish in a broad range of environmental circumstances, such as various wastewater types [39–43] They can also reduce soluble biodegradable organic matter when it happens concurrently with the mixotrophy process [44, 45]. This quick and reversible process can be used for both living and dead biomass because it is not dependent on the metabolism of the microalgae [39, 46].

Microorganisms known as microalgae are extremely crucial in the biotechnology field. They can flourish in wastewater [47], such as LW because they are tolerant to high ammonium contents. The properties of microalgae species differ greatly, including cell composition, tolerance to harmful substances, adaptability, and efficiency in removing nutrients [48]. For providing the nutrients needed by microalgae for growth, LW has been seen as a sustainable substitute [49, 50]. As a result, there is a lot of interest in microalgae-based wastewater treatment (MbWT) as a possible and affordable replacement for LW treatment [51–53]. Because of the microalgae’s photosynthetic activity, MbWT can remove the phosphate (P) and nitrogen (N) found in LW with little energy expenditure, fixing significant volumes of CO_2_ from the atmosphere in this process [54–56]. Also, the production of proteins from microalgae biomass is considered a relevant point [45, 57]. The present work describes the most adapted fungal, yeast, and microalgal strain utilized for Livestock wastewater treatment, which started with fermentation and saccharification and bioethanol production using fungi such as *P. chrysogenium Cla* and *S. cerevisiae Sc* and also the application of algae *Chlorella vulgaris* in the treatment process.

## Materials and methods

### Collection of livestock wastewater

Collection of livestock wastewater samples was carried out from European rural farms in the Arab Republic of Egypt on the desert road linking Cairo and Alexandria in the desert hinterland of Giza Governorate. Following collection, the samples were coded, and stored at 4^o^C at the Hydrobiology Lab, National Research Center in the proper containers, until further analysis [58].

### Collection of marine samples for isolation of associated fungi

Marine sample collection was performed from different locations during June/2023 from Hurghada governate, Egypt. The samples include Hurgada sea water site1, Hurgada sea sediment, Hurgada sea water site2 Sponge, Hurgada sea water site1, Hurgada sea water, and Hurghada Abo monqar island seawater. Following the collection, the marine samples were coded, photographed, and stored at 4^o^C until further processing [59].

### Isolation of associated fungi

Using potato dextrose agar (PDA; potato extract 4 g, dextrose 20 g, sea salt 24.4 g, agar 20 g, distilled water up to 1 L, pH 6), endophytic fungi were isolated from the gathered marine samples, including water and sediments [59]. According to [60], the surface sterilization approach has been employed to isolate the sponge sample. In brief, the sponge tissue was chopped into tiny pieces (about 1 cm x 1 cm) and repeatedly cleaned with sterile seawater to get rid of any adhered debris. The sponges were soaked in 70% ethanol for 60 to 120 s to sterilize their surface, They were then repeatedly rinsed in sterile saltwater and dried with sterile cotton cloth. After being sterilized, the sponge tissue pieces were aseptically put on a Petri dish filled with Sabouraud Dextrose Agar (SDA) media prepared in 30 ppt seawater. The plates were then incubated for 7 days at 28^o^C and observed for fungal growth around the sponge implant [61].

### Decomposition and saccharification of livestock waste cellulosic biomass via fermentation by isolated fungi

Preparation of cellulosic biomass of livestock wastewater samples for fermentation by fungi including separation of the collected samples into two forms cellulosic biomass and livestock wastewater. Preparation of fermentation media was started by adding 25 g of cellulosic biomass and 25 ml of dist H2O in a 250 ml flask. The prepared flasks were autoclaved, inoculated with fungal spore suspension, and incubated for 10 days at 29^o^C [62, 63].

### Enzymatic and saccharification assessment

Enzymatic saccharification of livestock waste cellulosic biomass was carried out by mixing 1 ml of the fermented culture after filtration with 1.5 mL of 1% 3,5-dinitrosalicylic acid (DNS) The mixture was then heated for 15 min [64], demonstrated how reducing sugars were computed calorimetrically in these experiments with glucose serving as a standard. The absorbance was measured at 540 nm. The amount of enzyme that releases 1 mol of reducing sugars (measured as glucose) per ml per minute is known as cellulase activity [65]. According to Miller [64], (DNS) is a reagent made up of many components. It was synthesized as follows: Dissolve 200 g of Rochell salt (sodium potassium tartrate), 2 g of phenol, 0.5 g of sodium sulfite, and 10 g of 3, 5-dinitrosalicylic acid in 500 mL of 2% NaOH, then dilute to 1 L [66].

### Identification of the most potent fungal endophytes

Regular cultural, morphological, and microscopical traits were used to identify endophytic fungal isolates.

### Phenotypic identification of the selected fungus

To study the phenotypic characters of the selected potent fungi, the selected fungi were cultured on a potato dextrose agar plate and incubated for 7 days at 30^o^C. The plates were visualized and checked each day. The colony color, pigmentation, and mycelia were observed using a light microscope with magnification power 100. Identification was carried out based on current universal [67–70].

### Genotypic identification of the selected fungus

Using the Qiagen DNeasy Mini Kit and the manufacturer’s instructions, genomic DNA extraction was used to carry out the molecular identification of the chosen fungal strains. Two primers, ITS4 (5′-TCCTCCGCTTATTGATATGC-3′) and ITS5 (5′-GGA AGT AAA AGT CGT AAC AAG − 3′), were used for the PCR amplification. The reaction mixture was as follows: PCR temperature profile: 94 °C for 5 min, followed by 35 cycles of 94 °C for 30 s, 55 °C for 30 s, 72 °C for 90 s, and a final extension step at 72 °C for 5 min. 1 µg fungal genomic DNA, 1 µL (20 µM of each primer), 10 mM dNTPs mixture, 2 units of Taq DNA polymerase enzyme, and 10 µL 5× reaction buffer. ThermoFisher Scientific’s JeneJET purification kit was used to purify the PCR product, which was then sent to Macrogen in South Korea for sequencing [71]. BLAST, which is accessible in the NCBI database (GenBank C, https://www.ncbi.nlm.nih.gov/Genbank/National Institute of Biotechnology Information, Bethesda, Maryland, USA), was used to align the 18 S rRNA gene sequence [59].

### Ethanol production and estimation using *Saccharomyces cerevisiae*

#### Saccharomyces cerevisiae

The isolate *S. cerevisiae* was acquired from the culture collection of the microbial genetics department, NRC. The isolate was identified morphologically and genetically to confirm its identity.

#### Bioethanol production

Utilizing fermented animal waste cellulosic biomass—which contained a sugar solution produced by an enzymatic saccharification method—bioethanol was produced in a semi-liquid state throughout the fermentation process. To create the S. cerevisiae seed broth, a loop of the organism was inoculated into YEPD (yeast extract peptone dextrose broth medium), which comprises the following ingredients: 5 g L-1 yeast extract (Duchefa Biochemie, Netherlands), 5 g L-1 peptone (Daejung, Korea), 20 g L-1 xylose (Junsei, Japan), 1 g L-1 MgSO4 (Shinyo Chemical, Japan), 1 g L-1 KH2PO4 (Sigma, USA), and 16 g L-1 agar. and a 24-hour incubation period at 28 °C and 200 rpm for the culture material. till the growth reached 5 × 108CFU/mL. The following was the composition of the fermentation media: 100 ml of sterile distilled water and 100 g of fermented cellulosic biomass a sugar solution made from the enzymatic saccharification of cellulosic biomass combined in a 500 ml flask. The flasks were sterilized and then inoculated with 1 mL of cultures of saccharomyces seed broth. For 7 days, the inoculated flasks were incubated at 30 °C on a 200 rpm rotary shaker. Using GC-mass analysis, the amount of ethanol was ascertained following a fermentation period of 7 days [72]. 

#### Detection of the bioethanol by gas chromatography

At the Central Laboratories Network, National Research Center, and Cairo, Egypt, ethanol was detected using a GC model 7890B from Agilent Technologies, which was outfitted with a flame ionization detector. A PoraBOND Q column with an internal diameter of 25 m x 320 μm and a film thickness of 5 μm was utilized to achieve separation. Hydrogen was used as the carrier gas in the analyses, with a split-1:100 mode flow rate of 2.0 ml min-1, a 1 µl injection volume, and a temperature program of 150 °C for 10 min. At 250 °C, the injector and detector (FID) were maintained, respectively. Detector gases: air 300 ml min-1, hydrogen 30 ml min-1, and nitrogen make-up gas 25 ml min-1 [73, 74]. 

#### Scanning electron microscope (SEM)

saccharification ability and bioethanol production were investigated with a scanning electron microscope (SEM). The SEM was run on an increasing voltage range of 200 V to 30 kV and an operating voltage range of 5 to 30 kV. Before the exams, Using an Edwards S150A Sputter Coater, the ready samples were gold-sputter coated [75]. 

#### Microalgal strain selection for bioremediation of fungal-pretreated livestock wastewater

To identify the most suitable microalgal strain for growth in biologically treated livestock wastewater. A batch culture bioassay was conducted using Nile River algae exposed to various dilutions of the pretreated wastewater.

#### Microalgal collection and inoculum preparation

Nile River water was collected in a sterile 5 L container; the collected water sample was then centrifuged to concentrate all present microalgal taxa. An experimental batch culture system was established using continuously aerated 1 L conical flasks.

**Fig. 1:**
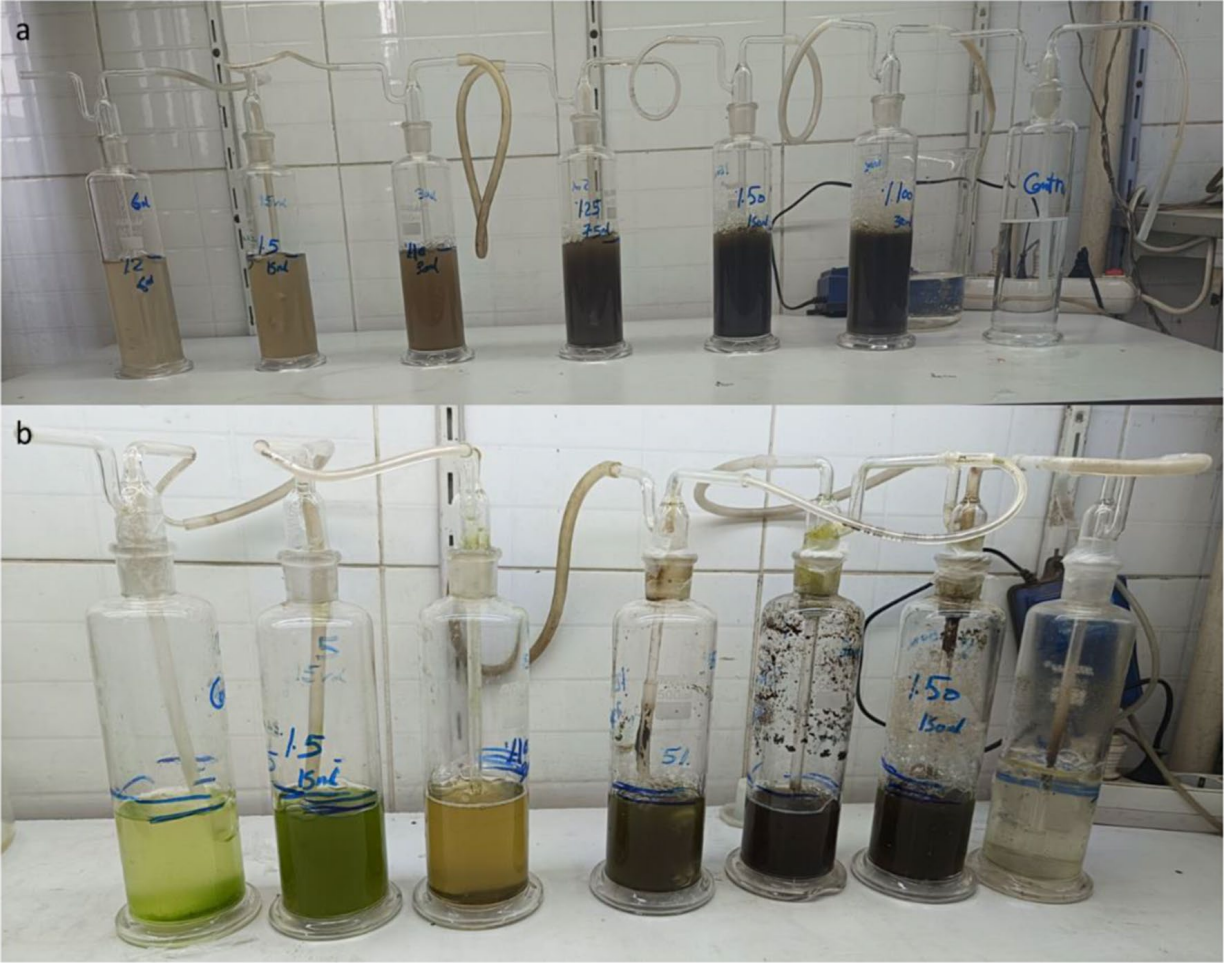
Bioassay experiment of the community composition demonstrates (**a**) the first and (**b**) the final incubation period

Two sets of flasks were prepared: (1) Control flasks containing distilled water supplemented with microalgae to achieve an initial chlorophyll a (chl. a) concentration of approximately 27 µg L-1. (2) Test flasks containing a mixture of diluted livestock wastewater (varying concentrations; see below) and microalgal inoculum. The final volume of the culture medium in each test flask was maintained at 500 mL, as shown in Fig. 1.

#### Wastewater dilution series and growth conditions

The group of pretreated livestock wastewater samples was diluted with distilled and another group was diluted with low-loaded mixed livestock wastewater to create two groups of series concentrations for testing: 2%, 5%, 10%, 25%, 50%, and 100%. The entire algae culture system was illuminated with cool white fluorescent lights providing an intensity of 750 lx. Room temperature was maintained throughout the experiment.

This methodology facilitates the evaluation of microalgal growth and tolerance across a range of wastewater concentrations, ultimately aiding in the selection of the most efficient strain for bioremediation applications utilizing fungal-pretreated livestock wastewater.

### Microalgal community analysis in fungal-pretreated livestock wastewater

#### Microscopic examination and species identification

Following the incubation period, samples from each culture flask (different wastewater concentrations) (Fig. 1) will be examined under a research microscope to assess the microalgal community. Sample Collection and Preservation: A small volume of culture will be collected from each flask. Lugol’s iodine solution will be used to preserve the algal cells and prevent degradation, Cell Counting, and Enumeration: Subsamples will be dispensed into a Sedgwick-Rafter counting cell, a specialized chamber designed for phytoplankton enumeration. The cell will be examined using an OLYMPUS CX41 microscope to identify and quantify the various microalgal taxa present [76]. Species Composition and Dominance: The relative abundance of each identified species will be determined, allowing for the calculation of dominance within the overall microalgal community. This will be done using a semi-quantitative approach. Taxonomic Identification: Algal species will be identified using established phytoplankton identification references, such as “Freshwater Algae of North America” [77]. This microscopic analysis will provide valuable insights into the impact of different wastewater concentrations on the microalgal community structure within the bioassay experiment.

#### The Treatment efficiency of nutrient and organic load after fungi-coded Cla, yeast-coded Sc, and algal treatment

The nutrient and organic load characteristics of the raw livestock wastewater sample were measured using standard methods outlined in APHA 2017. The raw sample was then subjected to treatment with the fungal strain (Cla) and the yeast strain (Sc), and the nutrient and organic load characteristics of the treated sample were measured using the corresponding APHA 2017 standard methods. The treatment efficiency, expressed as the percentage removal for each parameter, was calculated. The raw wastewater sample was also diluted with 5% organic load, and the nutrient and organic load characteristics of the diluted sample were measured according to APHA 2017. The diluted sample was then inoculated with microalgae from the Nile River, with a focus on *Chlorella vulgaris*, and the nutrient and organic load characteristics of the treated sample were measured using the relevant APHA 2017 standard methods, with the treatment efficiency calculated as the percentage removal for each parameter [78]. 

## Results and discussion

### Collection of livestock wastewater

**Fig. 2:**
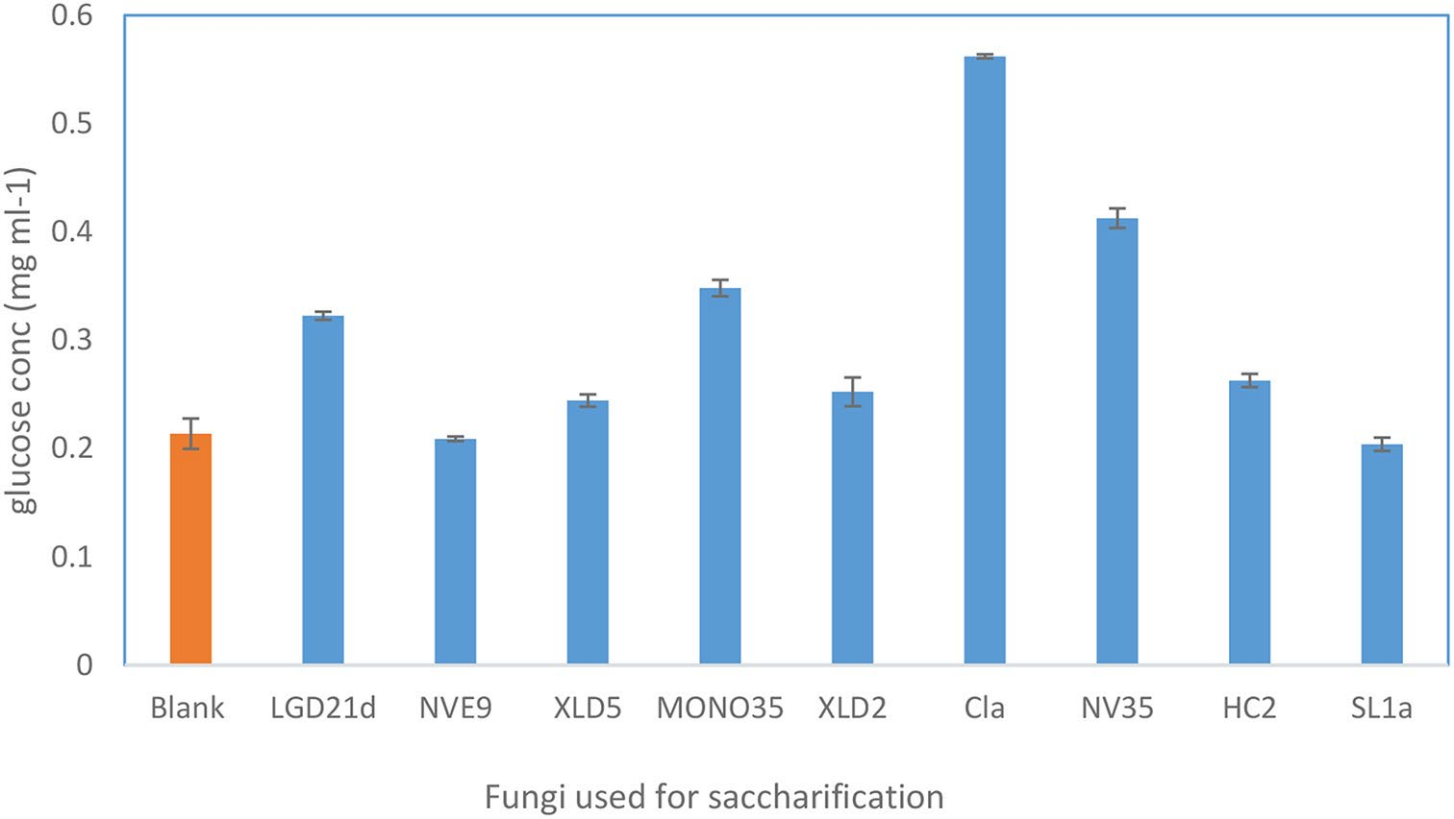
Detection of reducing sugar using (DNS) after saccharification using isolated fungi, the fig showed that Cla was the potent isolate

Collection of livestock wastewater was performed from the European rural farms, in Egypt, during May/2023 on the desert road linking Cairo and Alexandria in the desert hinterland of Giza Governorate (Fig. 2). The sample was taken from livestock, the livestock wastewater samples were collected, and preserved, until further processing.

### Marine sample collection and isolation of associated fungi

The marine sample was collected from different locations during June/2023, from Hurghada governate, Egypt. As demonstrated in the Table 1 Ten fungal strains coded as (Lgd21d, **Nve9**, Xld5, Mono35, Xld2, Cla, Nv35, Hc2, Sl1a, Nvef2) have been isolated using potato dextrose media [61]. According to recent studies, marine habitats, with their rich biodiversity, are ideal sources for isolating associated fungi. The endophytic mycobiota of analogous host species exhibit host specificity due to a variety of factors, including host species, host genotype, tissue origin, geography, nutrient availability, interactions with the host, and other abiotic and biotic stresses [79]. Around 9000 species of Porifera (sponges), 11,000 species of Cnidaria (jellyfish, corals, and sea anemones), 12,000 species of Mollusca, 7000 living and 13,000 extinct species of Echinoderms, Arthropoda (the most common phylum in the taxonomic system), as well as 70 species of mangrove plants that live in marine environments are among the various marine invertebrates surveyed by fungal research. These findings suggest the ubiquity of endophytes and demonstrate their symbiotic relationships in all healthy taxa that have been studied to date [79–83].

### Decomposition and saccharification of livestock waste cellulosic biomass

Enzymatic hydrolysis of livestock waste cellulosic material by fungal cellulosic enzymes has recently attracted researcher’s attention and it is considered one of the most promising approaches. Studies showed that extracellular cellulase enzymes, produced by fungi, may quickly decompose cellulose into two or three glucose units, which can then be easily absorbed as glucose monomers [84]. Filamentous fungi produce a variety of enzymes that break down the polysaccharides found in plant cell walls which the food and feed industries depend on [85–88]. 

The isolated marine-derived fungi were screened for their ability to ferment and hydrolyze the livestock waste which contains polysaccharide and cellulosic materials (Fig. 2). The process was started by autoclaving the livestock waste and inoculation of each obtained fungus separately. After incubation, As previously noted, the DNS approach was used to determine the overall quantity of reduced sugar. The results of the screening experiment reveal the potential of marine-derived fungi to ferment and hydrolyze livestock waste containing polysaccharides and cellulosic materials. The process involved autoclaving the livestock waste followed by inoculation with each isolated fungus separately. After the incubation period, the concentration of reduced sugar, primarily glucose, was determined using the DNS technique. The average concentration of reducing sugar varied among the different fungal isolates, indicating variations in their ability to degrade and ferment the complex polysaccharide substrates present in livestock waste. Among the tested fungi, Cla exhibited the highest average concentration of reducing sugar at 0.56 mg ml^− 1^, suggesting its robust capability to hydrolyze polysaccharides and convert them into simpler sugars such as glucose. Comparatively, Lgd21d, Mono35, and Nv35 also demonstrated relatively high average concentrations of reducing sugar, indicating their effective fermentation and hydrolysis abilities. In contrast, Control sample and fungi such as Nve9, Sl1a, and Nvef2 exhibited lower average concentrations of reducing sugar, suggesting potentially lower efficiency in degrading the polysaccharide substrates present in the livestock waste.

### Determine the amount of bioethanol through fermentation by yeast in a mixed sample inoculated by fungi and yeast

#### Phenotypic identification of fungi coded (Cla, Nv35, Mono35)

**Fig. 3:**
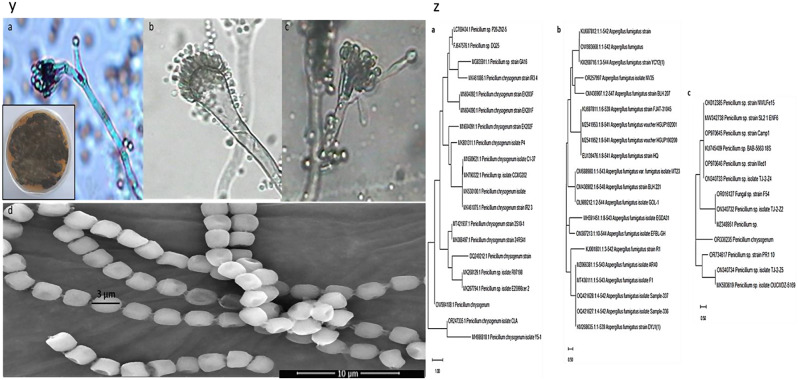
(**y.a**) Light microscopic examination and colony morphology of Cla isolate (**y.b** Nv35 isolate (**y.c**) Mono35 isolate and (**y.d**) scanning electron micrograph of Cla, (**z.a**) Constructed phylogenetic tree for *Penicillium chrysogenum Cla*, (**z.b**) *Aspergillus fumigatus* spp Nv35, (**z.c**) *Penicillium chrysogenum Mono35*

The examination of three isolates reveals distinctive growth characteristics and microscopic features, providing valuable insights into their taxonomy and potential applications. Figure 3(y) shows the microscopic examination of the three selected fungi. (Fig. 3y.a) isolate **(Cla)** displayed colony morphology on Potato dextrose agar media, while the microscopic examination showed the following characteristics: Colonies on Czapek Yeast Extract Agar (CYA) display a diameter of 2–3 cm at 25 °C, exhibiting colors ranging from white to grayish, with mycelium appearing deep green. The reverse side of colonies appears pale yellow to brown. Notably, microcolonies exhibit growth on CYA at both 5 °C and 37 °C, indicating adaptability to different temperature ranges. Microscopically, conidiophores may be either mono or bi-verticillate. The diameter of conidiophores measures approximately 2.5 μm. Metulae, the structures supporting phialides, are observed to be 14 μm in length and 2.3 μm in diameter, while phialides, the cells responsible for conidia production, measure approximately 7.0 μm in length and 2.0 μm in diameter. The conidia produced by Penicillium are spherical to sub-spherical, with a diameter of 2.5 μm. These microscopic characteristics aid in the identification and classification of Penicillium species [67]. Additionally, (Fig. 3y.b) isolate **(Nv35)**, showed rapid Colonies spreading on Czapek Agar and MEA at 25 °C, observation showed that it displayed Aspergillus-type conidiophores, the diameter of conidiophores measures approximately 6 μm. Conidial heads, the structures supporting Vesicles and Sterigmata, are observed to be 180 μm in length and 30 μm in diameter, while vesicles are fertile over the upper half only, and measure approximately 25 μm in diameter. While Sterigmata measures approximately 6 μm in length and 2.2 μm in diameter. The conidia produced by Aspergillus are globose, echinulate, and green-colored, with a diameter of 2.8 μm. These microscopic characteristics aid in the identification and classification of Aspergillus species. Moreover, the (Fig. 3y.c) isolate (**Mono35)** showed the same characteristics as the isolate Cla. Therefore, based on the microscopic examination, isolates Cla and Mono35 were found to belong to *Penicillium sp*, While isolate Nv35 belonged to *Aspergillus sp* (Fig. 3y. a, b, and c). Confirmation of the Cla identity as a potent isolate was carried out by Scanning electron microscopy (Fig. 3y.d) [67–70].

#### Genotypic identification of fungi coded (Cla, Nv35, Mono35)

The isolated fungus Fig. 3z (a, b,c) was genetically identified using sequencing methods that focused on the 18 S rRNA gene. Using the Basic Local Alignment Search Tool (BLAST), the extracted DNA was amplified, processed, and aligned with known sequences kept in the GeneBank database. The acquired results showed a great deal of similarity between the fungal isolates (a)(b)(c) and the acquired sequences, with a homology of 99.81%, 100%, and 95.73 corresponding to *P. chrysogenum* spp.,* A. fumigatus* spp., and *P. chrysogenum*spp. Similarly, the fungal isolates have been archived in GenBank under the accession numbers OR247335, OR257997. and OR336235 respectively. The evolutionary history is deduced by using the neighbor-joining method, as suggested by [89]. This is the optimal tree. The percentage of duplicate trees in which the taxa were grouped together was calculated using the bootstrap test [90] and is shown next to the branches. The tree has been accurately scaled so that the lengths of the branches correspond to the evolutionary distances used in the phylogenetic tree inference (Fig. 3z) The evolutionary distances were calculated using the Maximum Composite Likelihood technique and are expressed in units of the number of base substitutions per site [91]. There were 20, 20, and 13 nucleotide sequences in the current study. An investigation of the first, second, third, and noncoding locations of the codons was included in this study. For every pair of sequences, the paired deletion option was used to eliminate any instances of unclear placements. There were 1755 locations in the final dataset. We used the program MEGAX to do evolutionary analysis [92, 93].

#### Obtaining and identification of *Saccharomyces cerevisiae*

**Fig. 4:**
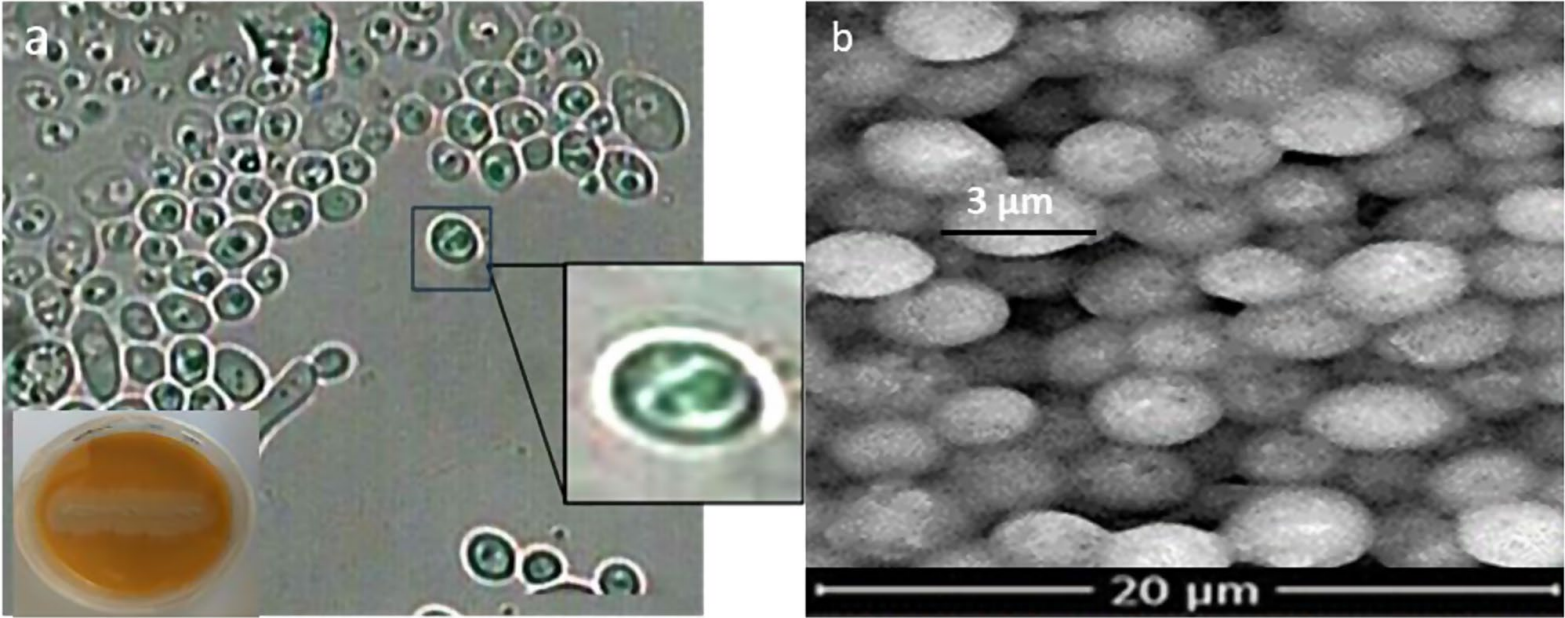
(**a**) Light microscope examination (**b**) scanning electron microscope of *Saccharomyces cerevisiae*

In an industrial context, *S. cerevisiae* outperforms bacteria, other yeasts, and filamentous fungi in several physiological characteristics related to ethanol production, such as the capacity for fermentation and aptitude to cope with harsh environmental conditions like high ethanol and organic acid concentrations, low pH levels, limited oxygen availability, and nutrient depletion [94–96] Unquestionably, *S. cerevisiae* is one of the most fermentative-prone microbial species, which can produce ethanol even in the presence of excess of oxygen (Crabtree effect) and exhibiting fast rates of sugar consumption and ethanol production [97] Additionally, this species is tolerant of high ethanol and organic acid concentrations [95, 98]. and can grow and ferment sugar at the low pH values (3.0–3.5) of grape musts. *S. cerevisiae* is also one of the few yeast species that can thrive under strictly anaerobic conditions [97], having low nitrogen requirements [99–101], It is less prone to fermentation infection than bacteria. moreover, it is more ethanol-tolerant than other microorganisms that produce ethanol [102]. Since *S. cerevisiae* is GRAS (generally regarded as safe) for human consumption, it can be used more advantageously than other yeasts and microbes [103]. The *Saccharomyces* isolate was acquired from the culture collection of the microbial genetics department, NRC. The examination of *Saccharomyces (Sc)* isolates reveals distinctive growth characteristics and microscopic features, providing valuable insights into its taxonomy and potential applications. Colonies produced on culture plates are typically creamy white discs with well-defined circular edges. Individual cells or small clusters with oval or round shapes and diameters ranging from 3 to 8 micrometers are visible under a microscope. Unlike some other yeast species, *S. cerevisiae* does not produce pseudohyphae (elongated filaments) or chlamydospores (thick-walled resting spores). These combined morphological and microscopic traits are a useful tool for identifying *S. cerevisiae* [70] (Fig. 4a and b).

#### Genotypic identification of yeast

**Fig. 5:**
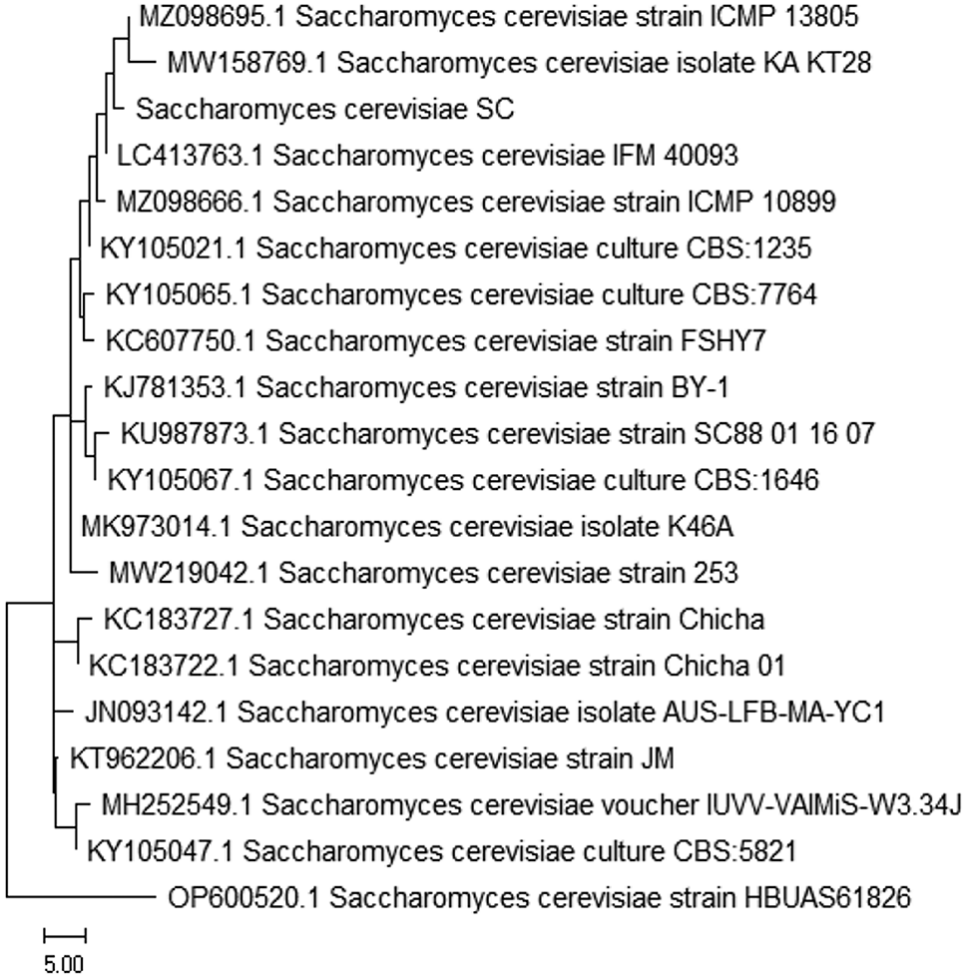
Constructed phylogenetic tree of *Saccharomyces cerevisiae (sc)*

The isolated yeast Sc was genetically identified using sequencing methods that focused on the 18 S rRNA gene. Using the Basic Local Alignment Search Tool (BLAST), the extracted DNA was amplified, processed, and aligned with known sequences kept in the GeneBank database. The acquired results showed a great deal of similarity between the yeast isolate Sc and the acquired sequences, with a homology of 99.40% corresponding to *S. cerevisiae*spp. Similarly, the fungal isolates have been archived in GenBank under the accession number PP859871. The evolutionary history is deduced by using the neighbor-joining method, as suggested by [89]. This is the optimal tree. The percentage of duplicate trees in which the taxa were grouped was calculated using the bootstrap test [90] and is shown next to the branches. The tree has been accurately scaled so that the lengths of the branches correspond to the evolutionary distances used in the phylogenetic tree inference (Fig. 5) The evolutionary distances were calculated using the Maximum Composite Likelihood technique and are expressed in units of the number of base substitutions per site [91]. There were 20 nucleotide sequences in the current study. An investigation of the first, second, third, and noncoding locations of the codons was included in this study. For every pair of sequences, the paired deletion option was used to eliminate any instances of unclear placements. There were 1755 locations in the final dataset. We used the program MEGAX to do evolutionary analysis [92, 93]. 

### Saccharification and bioethanol production

**Fig. 6:**
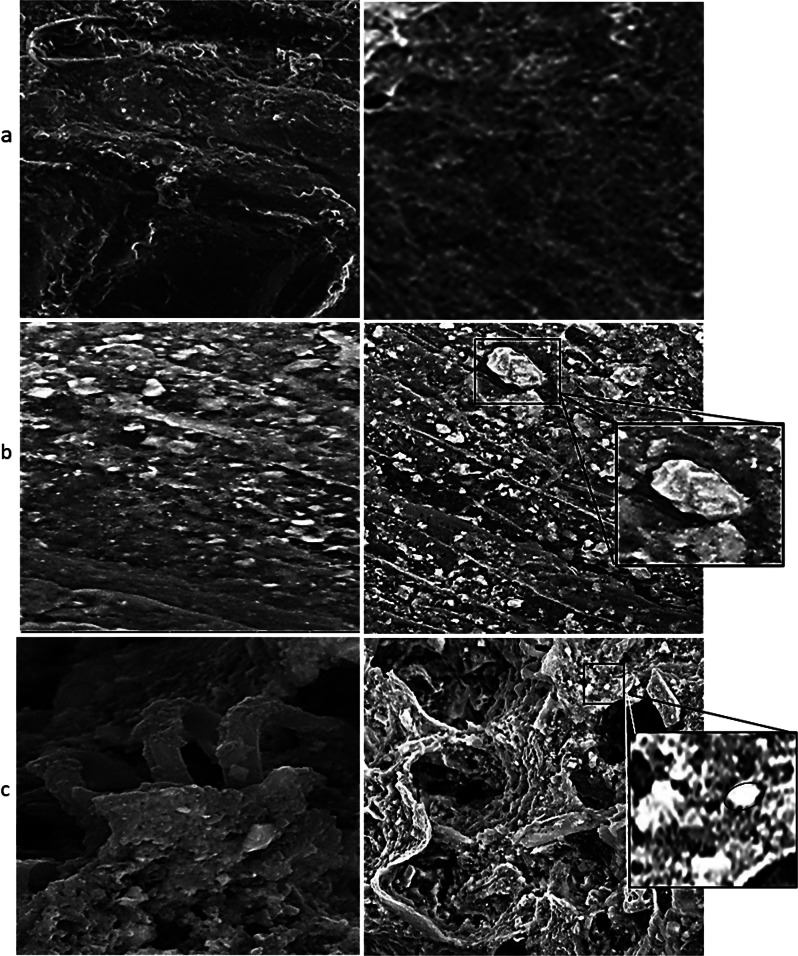
(**a**) Waste sample without treatment with fungi and yeast, (**b**) waste sample after treatment with *P. chrysogenum cla*, and (**c**) Waste sample after treatment with *P. chrysogenum cla* and *S. cerevisiae Sc.*

The saccharification ability of the three fungi and yeast was studied by fermentation on the cellulosic biomass for a certain period. The treatment condition was as follows: waste without treatment, waste inoculated with *P. chrysogenum* Cla and then *S. cerevisiae Sc*, waste inoculated with *A. fumigatus Nv35* and then *S. cerevisiae* Sc, and *P. chrysogenum Mono35* and then *S. cerevisiae Sc*. Analysis of the Saccharification and ethanol production process was carried out via scanning electron microscope and GC-mass analysis. Figure 6 shows the difference between the waste without treatment, and waste after treatment with *P. chrysogenum Cla* and waste after treatment with *P. chrysogenum Cla* and then *S. cerevisiae Sc*. Figure 6a displays the undigested organic matter such as fibers, remnant plant material, or complex carbohydrates. While Fig. 6b shows the fungal hyphae growth (filaments) with a characteristic branching pattern. Possible adhesion of fungal hyphae to organic matter particles, suggesting initial stages of degradation. Figure 6c shows the waste after yeast Inoculation (*Saccharomyces cerevisiae*) which is typically spherical or ellipsoidal in SEM images. Possible signs of yeast attachment to organic particles or fungal hyphae suggest collaborative biodegradation.

Table 2 shows the results of bioethanol production from livestock waste via fermentation with different treatments, including *P. chrysogenum Cla* and *S. cerevisiae Sc*, *A. fumigatus Nv35* and S. cerevisiae Sc, and *P. chrysogenum Mono35* and *S. cerevisiae Sc*, The area under the peak and the concentration of produced ethanol in mg ml^− 1^ are provided for each treatment. Upon analyzing the results, it is evident that the combination of *P. chrysogenum Cla* with *S. cerevisiae Sc* yielded the highest area under the peak (161669.52), followed by *P. chrysogenum Mono35* and S. cerevisiae Sc (115449.04), then, *A. fumigatus Nv35* and *S. cerevisiae Sc* (96978.99). This indicates that, in comparison to the other treatments, the presence of *P. chrysogenum Cla* greatly increased the synthesis of bioethanol. Furthermore, when considering the concentration of produced ethanol in mg ml-1, it is notable that the highest concentration observed is the *P. chrysogenum Cla* and *S. cerevisiae Sc* (0.10 mg ml -1), followed by *P. chrysogenum Mono35* and *S. cerevisiae Sc* (0.07 mg ml-1), and *A. fumigatus Nv35* and *S. cerevisiae Sc* (0.06 mg ml-1). These results confirm the trend observed beneath the peak, further supporting the superior bioethanol production capacity of the *P. chrysogenum Cla* with *S.cerevisiae Sc* treatment.

**Fig. 7:**
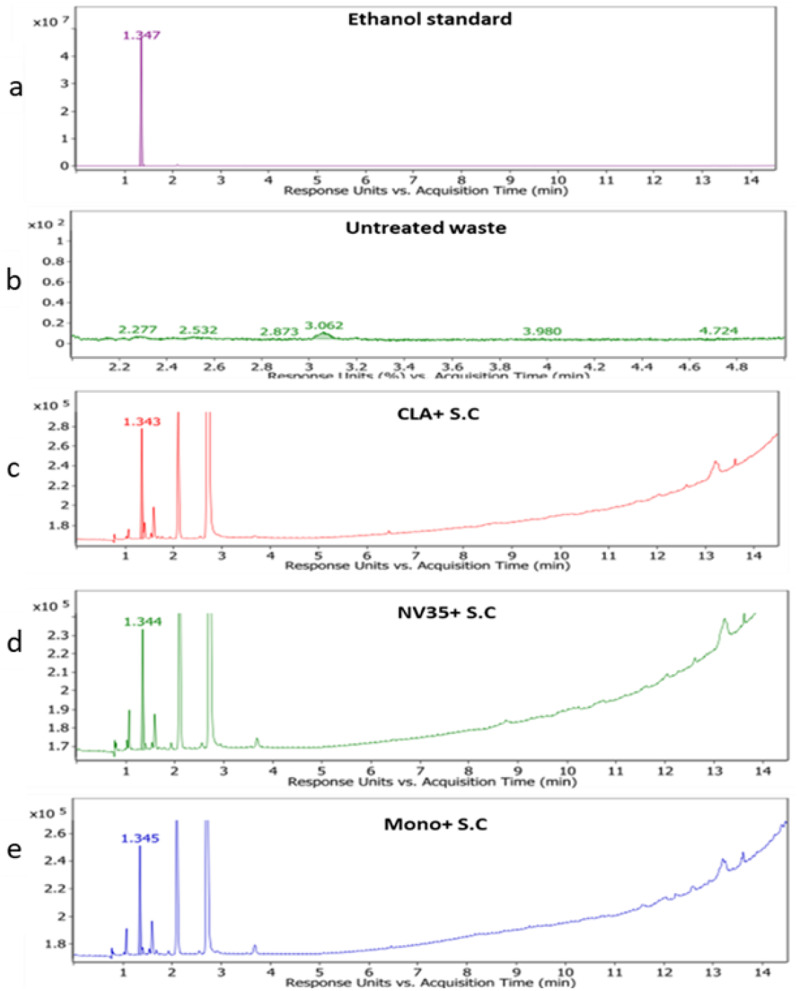
Detection of bioethanol production by different treatments while (**a**) represent the Standard Ethanol, (**b**) untreated waste, (**c**) *P. chrysogenum* Cla and *S. cerevisiae Sc*, (**d**) *A. fumigatus Nv35* and *S. cerevisiae Sc* and (**f**) *P. chrysogenum* Mono35 and *S. cerevisiae Sc*

It is significant to note that, as shown in Fig. 7, ethanol was utilized as a standard for GC analysis, guaranteeing precise measurement of the ethanol concentrations in the samples. Overall, the findings imply that, in comparison to previous treatments, the combination of *P. chrysogenum Cla* and *S. cerevisiae Sc* improves bioethanol production from livestock waste fermentation. This demonstrates how using fungal strains in conjunction with *S. cerevisiae* can increase the yield and efficiency of bioethanol synthesis in biofuel operations. Further studies could explore the fundamental mechanisms behind the synergistic impacts of fungal strains on bioethanol production and optimize fermentation conditions for augmenting ethanol yields.

### Impact of organic load on microalgal community composition

**Fig. 8:**
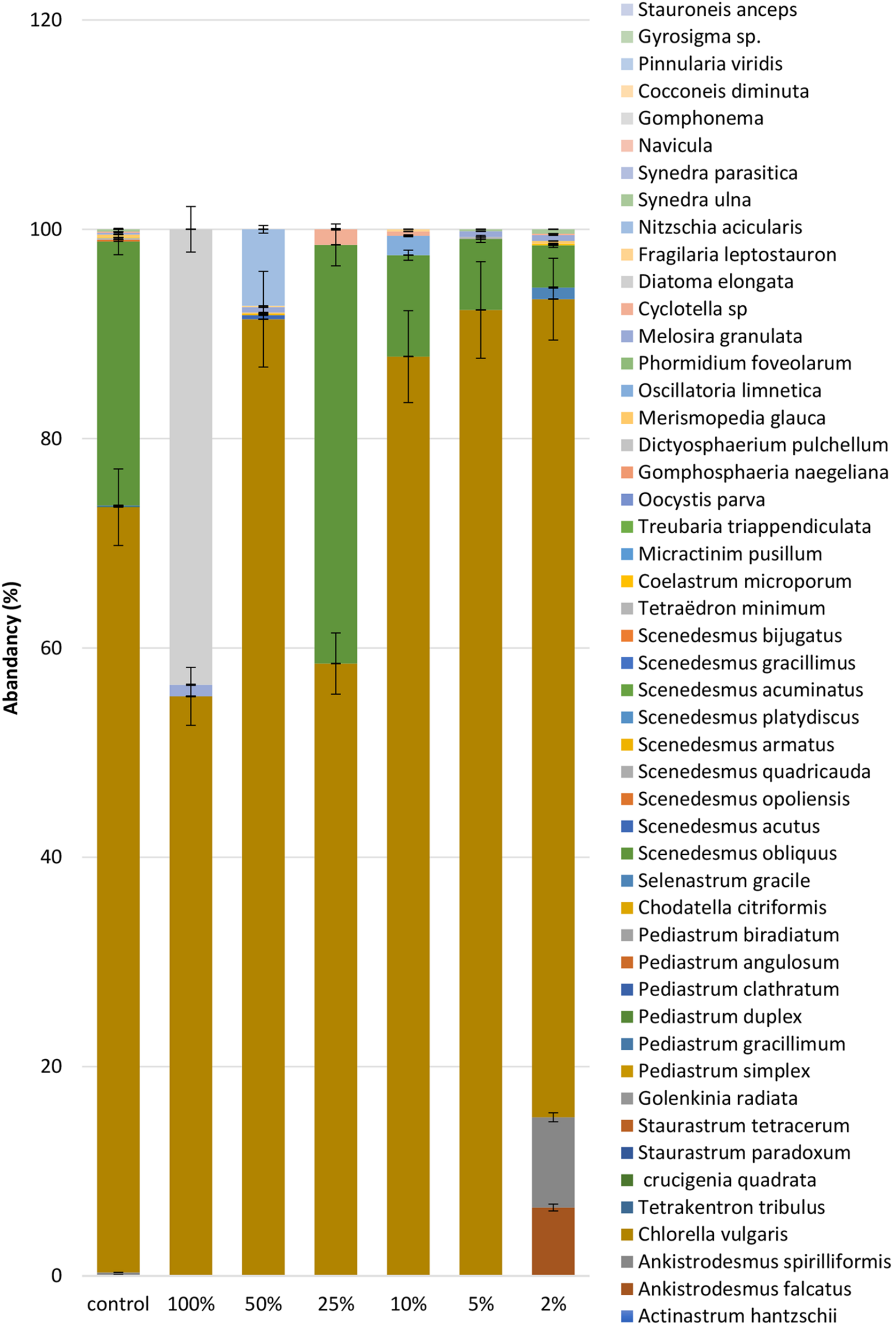
Impact of organic load on dominant microalgal species in diluted livestock wastewater

**Fig. 9:**
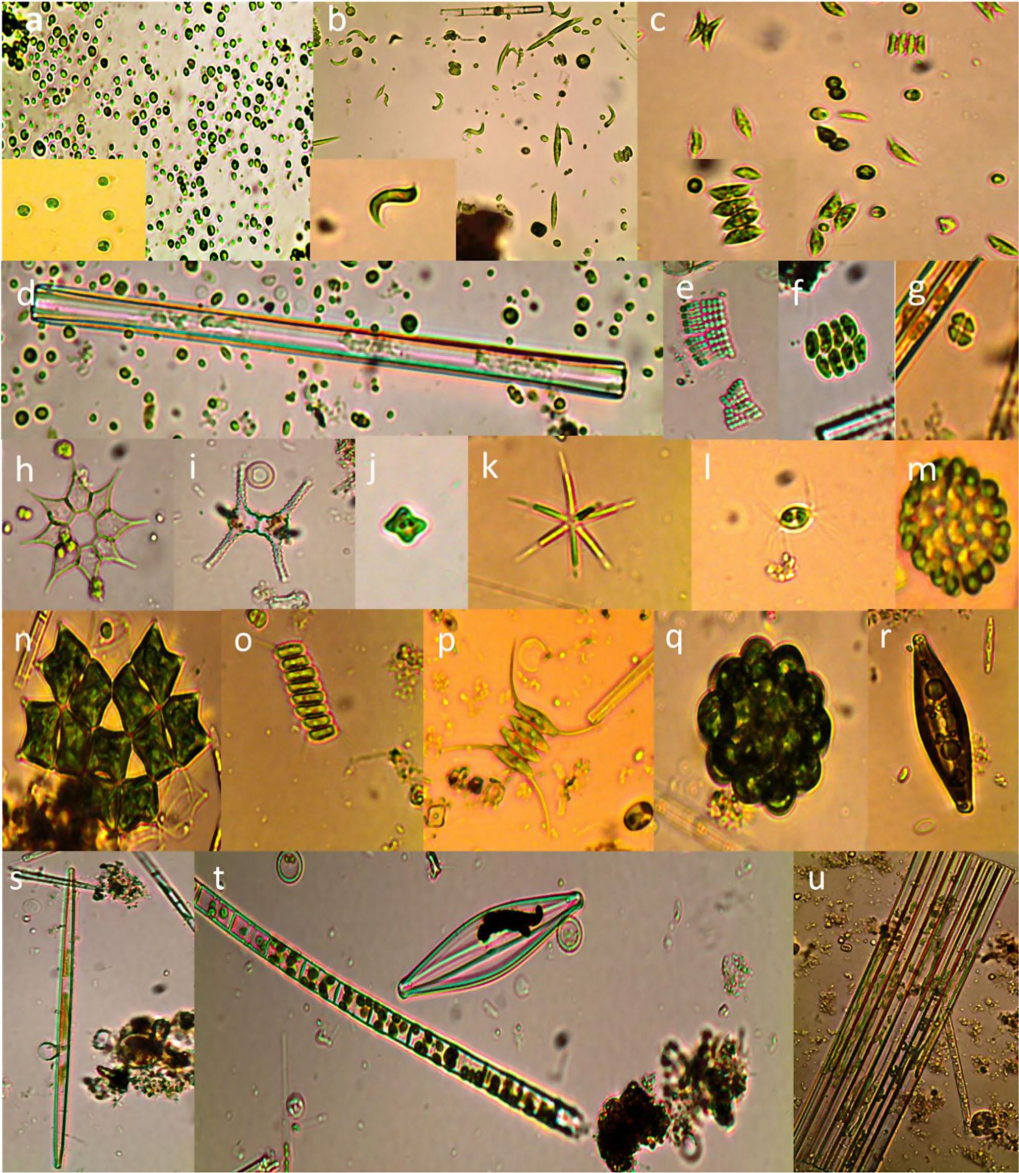
Representative images of microalgal species identified in diluted livestock wastewater. (**a**): *Chlorella vulgaris*, (**b**): *Ankistrodesmus falcatus*,* Ankistrodesmus spirilliformis* (**c**): *scenedesmus obliquus* (d): *diatoma elongate.*, (**e**): *Merismopedia glauca*, (**f**): *scenedesmus platydiscus*, (**g**): *crucigenia quadrata*, (**h**): *Pediastrum simplex*, (**i**): *Staurastrum* paradoxum (**j**): *Tetraëdron minimum*, (**k**): Actinastrum hantzschii, (**i**): *chodatella citriformis*, (**m**): *Dictyosphaerium pulchellum*, (**n**): *Pediastrum duplex*, (**o**): *Scenedesmus quadricauda*, (**p**): *scenedesmus opoliensis*, (**q**): *coelastrum microporum*, (**r**): *Navicula*, (**s**): *Synedra ulna*, (**t**): *Melosira granulate*, (**u**): *Fragilaria leptostauron*

As demonstrated in Figs. 8 and 9. The microalgal community exhibited a dynamic response to varying concentrations of organic load derived from diluted algal-inoculated livestock wastewater. We evaluated different organic load scenarios ranging from 2 to 100% dilution. At a 2% organic load, *Chlorella vulgaris* emerged as the most abundant species, constituting 73.1% of the microalgal community. Interestingly, *Chlorella vulgaris* maintained its dominance even as the organic load increased to 5% and 10%, with a slight decrease in relative abundance to 55.4% and 58.5% respectively. This finding is consistent with earlier studies demonstrating the adaptability of *Chlorella vulgaris* to different organic loads in wastewater treatment systems [104, 105]. However, a shift in the dominant species occurred at a 25% organic load, with *Scenedesmus obliquus taking* over at 40% abundance. This suggests a potential threshold for *Chlorella vulgaris* dominance, beyond which other species with higher tolerance to organic matter may become more competitive [106]. The resilience of *Chlorella vulgaris* was again evident at 50% organic load, where it regained dominance with a significant increase to 87%. This highlights the remarkable adaptability of this species across a variety of organic load conditions [107]. This dominance continued at the highest organic load tested (100% dilution), with *Chlorella vulgaris* comprising a remarkable 92.3% of the microalgal community. It’s important to note the presence of other species such as *Ankistrodesmus falcatus*, *Ankistrodesmus spirilliformis*, *Selenastrum gracile*, and *Melosira granulate*, although their contribution to the overall community was less substantial. This observed distribution pattern underscores the dynamic nature of the microalgal community in response to varying organic loads. *Chlorella vulgaris* stands out as the dominant species across most scenarios, demonstrating its remarkable adaptability and tolerance to diverse organic load conditions within livestock wastewater. According to the above data we calculated the Growth rate µ (d^− 1^) at each organic load concentration as the following equation: [108]

1 $$\mu =\ln (\text{B}_{\text{T}}/\text{B}_{0})/\text{T}$$

Where T is the unit time interval in days and B_T_ and B_0_ are the total algal count (org. ml) at the start (0) and end of the time interval (T).

### Optimizing microalgal growth rate through organic load variation

**Fig. 10:**
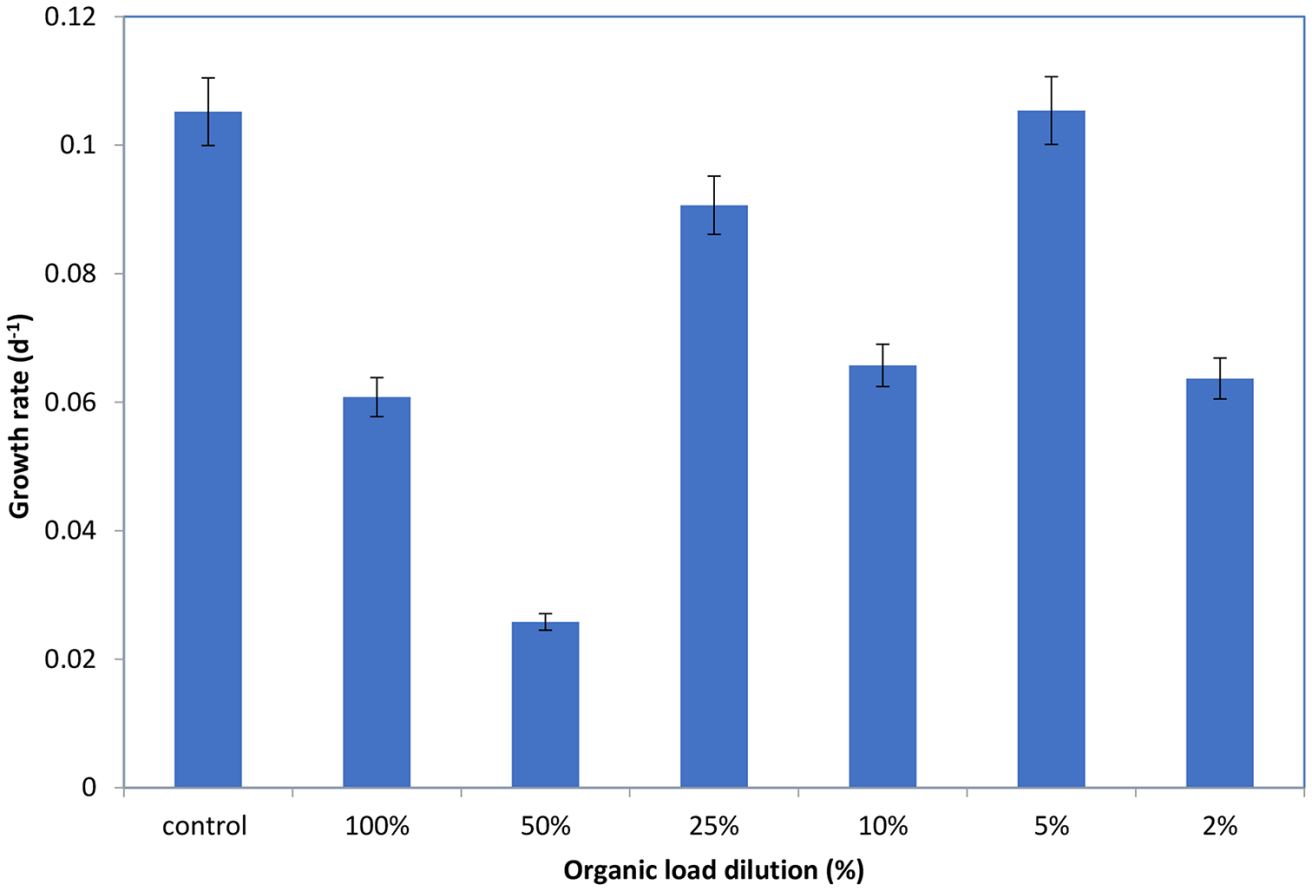
Effect of organic load on microalgal growth rate in diluted livestock wastewater

The data presented in Fig. 10 suggests a relationship between organic load concentration and microalgal growth rate in diluted algal-inoculated livestock wastewater. We investigated growth rates at various organic load concentrations: 2%, 5%, 10%, 25%, 50%, and 100%. This analysis aimed to identify the optimal organic load concentration for maximizing microalgal production.

Our findings revealed a distinct pattern in growth rate across the different organic load concentrations. At 2% organic load, the growth rate was moderate (0.063689), indicating a suitable but not optimal environment for microalgal growth [109]. Interestingly, the growth rate peaked at 5% organic load (0.105385), suggesting this concentration provided the most favorable conditions for microalgae to flourish [110]. A slight decrease in growth rate was observed at 10% organic load (0.065724), signifying a less ideal but still supportive environment. The growth rate displayed another notable increase at 25% organic load (0.090652), likely due to the higher availability of nutrients at this concentration. However, at 50% organic load, the growth rate dropped significantly (0.025804), suggesting limitations and challenges for microalgal growth under these conditions, potentially due to nutrient overload or the presence of inhibitory compounds [111]. Lastly, the growth rate at 100% organic load (0.06079), while higher than the 50% concentration, remained lower than those observed at lower load concentrations. This suggests that the high organic load may be causing the microalgae to go into stress. The investigation concluded that the concentration that yielded the maximum microalgal growth rate was 5% organic load. According to this research, a reasonable amount of organic load offers the best nutrient balance, allowing for promoting growth and reproduction. To maximize microalgal production, cultivation efforts should focus on maintaining a 5% organic load environment. However, To properly comprehend the underlying mechanisms causing this choice, more research is necessary. Several factors could be involved, including competition with other microbes, metabolic processes, and the availability of particular nutrients [112]. Understanding these factors can be crucial for refining cultivation strategies and optimizing microalgal production for diverse applications. For instance, One of these important aspects is nutrient availability, further studies could explore diluting the 5% organic load with particular nutrient mixtures derived from livestock wastewater. This strategy might potentially provide a more specialized nutritional environment to further promote microalgal growth.

The availability of nutrients is a crucial component in the growth of microalgae [113]. The approach comprised dilution of the 5% organic load with certain nutrient combinations derived from livestock wastewater to investigate this issue. Establishing a more tailored nutritional environment potentially further improves microalgal growth. The results show significant variations in the amounts of nutrients before and after the addition of livestock wastewater-derived nutrient mixtures. For instance, NH_3_ increased from 28.75 to 107.3, NO_2_ increased from 0.05 to 35.6, NO_3_ increased from 0.05 to 21.73, and PO_4_ increased from 10.55 to 32. According to [114], a balanced nutrient supply is necessary for the best microalgal growth rate and lipid productivity. These changes imply that the introduced nutrient mixtures were instrumental in increasing nutrient availability within the microalgal cultivation system. It is noteworthy that additional research is necessary to determine the precise mechanisms underlying the observed variations in nutrient concentrations and microalgal development. The overall results may be influenced by variables like nutrient uptake, metabolic activities, and possible competition with other microorganisms. As stressed by [115], comprehending these mechanisms is essential for fine-tuning cultivation techniques and maximizing microalgal output for various applications.

**Fig. 11:**
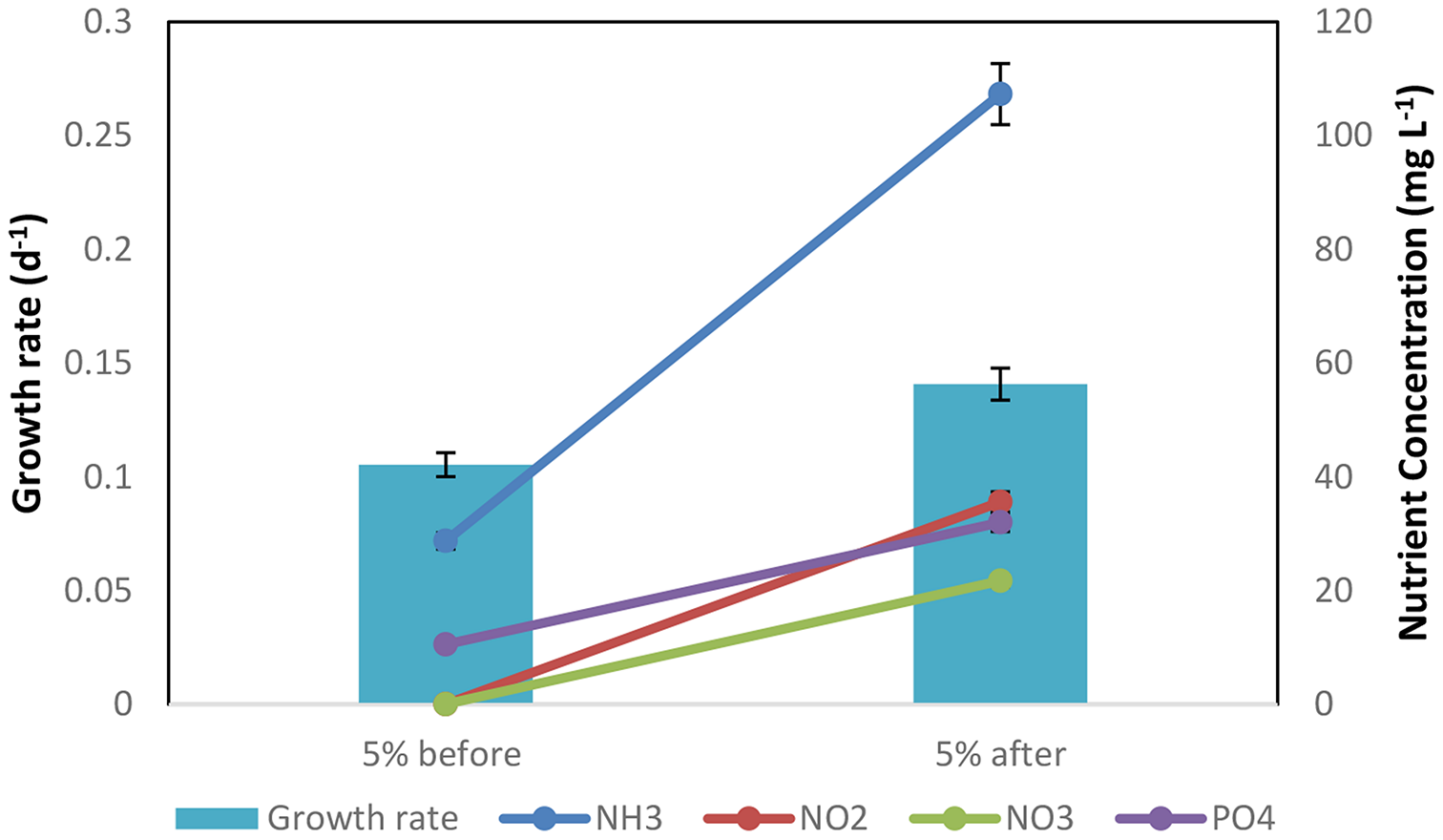
Impact of nutrient addition from livestock wastewater on microalgal growth rate

The analyzed data reveals significant differences in several parameters before and after the addition of nutrient mixtures derived from livestock wastewater (Fig. 11). Notably, with the addition of nutrients, the microalgae growth rate increased from 0.105385 to 0.140785. This result implies that the introduction of particular nutrient mixtures may have a beneficial effect on the development and reproduction of microalgae. The concentration that yielded the maximum microalgal growth rate, according to the investigation, was 5% organic load. This indicates that a reasonable amount of organic load provides an optimal nutritional balance, Therefore, to enhance microalgal production, it is advised that cultivation efforts concentrate on maintaining an environment with a 5% organic load. However, further investigation is necessary to completely understand the underlying mechanisms driving this preference, echoing the importance of research highlighted by [116] on finding the optimal organic load for microalgal cultivation using diluted wastewater [117]. investigated the viability of using livestock wastewater as a microalgal culture nutrient, achieving successful cultivation. Similarly [56], investigated the use of microalgae for the treatment of livestock wastewater, showing promise for biomass generation and nutrient recovery, thus advancing resource recovery and waste management. As seen by the enhanced growth rate, the results from Fig. 11 show that the addition of particular nutrient mixtures made from livestock wastewater can greatly promote microalgal growth. These results emphasize the significance of nutrient availability and imply that the optimal balance for optimizing microalgal production might be achieved at a concentration of 5% organic load. Further research is needed to unravel the underlying mechanisms and explore the potential of tailored nutrient mixtures to further enhance microalgal growth in a controlled environment, as highlighted by Mata et al. (2010) who discussed various factors affecting microalgal growth.

### The treatment efficiency of nutrient and organic load after *P. chrysogenum* spp. (Cla), *S. cerevisiae* (Sc), and algal treatment

The outcomes shown in Tables 3 and 4 show how well the fungi-yeast-microalgae consortium treats the nutrient and organic load characteristics of the livestock wastewater.

Table 3 displays the treatment effectiveness after the *P. chrysogenum* spp (Cla) and *S. cerevisiae (Sc)* treatment. The results show that this treatment was highly effective in removing phosphate (PO_4_), with a 63.2% reduction. However, there was no appreciable amount of removal of ammonia (NH3), nitrite (NO_2_), or nitrate (NO_3_). Additionally, The treatment demonstrated a moderate reduction in COD (43.8%) and a significant drop in BOD (80.8%). These results are in line with previous studies showing the effectiveness of fungi, especially *Penicillium* species, in breaking down organic pollutants and treating wastewater [118, 119]. Furthermore, the impact of nutrient valorization in wastewater treatment systems is demonstrated [120].

Table 4 shows the results of the algal treatment of the diluted wastewater. This stage of the treatment was highly effective, achieving a complete removal of phosphate (100%), a 94.1% reduction in nitrate, and a 92.5% reduction in ammonia. the algal treatment showed notable drops in COD (96.5%) and BOD (96.1%). These results are in line with earlier studies [121–123] that showed how effective algae treatment is in drastically lowering pollutants in wastewater. For example, these investigations showed significant and noteworthy decreases in ammonia, nitrate, phosphate, COD, and BOD.

These findings demonstrate the complementary roles of the fungi-yeast and microalgal treatments to address the different nutrient and organic load components of livestock wastewater. While the microalgal treatment was essential for removing the remaining nutrients and organic waste, the fungal-yeast treatment was successful in saccharification and the production of bioethanol.

. Similar to the synergistic study [124] that demonstrated the effects observed in *Desmodesmus*-*Klebsiella* co-cultures for tetracycline removal, fungi-yeast, and microalgae combinations can potentially enhance the treatment of livestock wastewater through complementary metabolic functions.

The integrated treatment process employing fungi, yeast, and microalgae demonstrated the potential for effective nutrient removal and bioethanol production from livestock wastewater, aligning with previous research on the synergistic interactions between these microorganisms [120, 125].

## Conclusion

In this study, we investigated a multi-phase strategy for tackling the challenges of limited renewable energy sources and livestock-polluted water. to achieve both wastewater treatment and bioethanol production, we utilized a consortium of microorganisms including fungi, yeast, and Nile water microalgae. First, we used a variety of fungal strains to investigate the saccharification process. The most effective fungus was *P. chrysogenum*, which showed an amazing capacity to break down complex organic materials found in livestock wastewater into simpler sugars, producing about 0.56 mg ml^− 1^ of glucose. Following this, the study turned its attention to yeast fermentation. The most effective strain of yeast was found to be *S. cerevisiae*, which was able to convert the sugars generated by the fungus into bioethanol at a rate of approximately 99.32 ppm. Lastly, we looked into treating the wastewater that had already been pretreated using Nile water microalgae. In a 5% concentration of the pretreated wastewater, *Chlorella vulgaris* microalgae showed the most encouraging growth, highlighting its potential for further wastewater treatment. These results suggest a promising multi-stage system utilizing a combination of fungi, yeast, and microalgae for not only treating livestock wastewater but also generating bioethanol as a valuable byproduct. Furthermore, *Chlorella vulgaris’s* ability to thrive in a 5% organic load environment underscores the potential for optimizing this process by balancing nutrient availability with the potential inhibitory effects of high organic matter content. results highlight the complementary roles of the fungi yeast and microalgal treatments in addressing the various nutrient and organic load components of livestock wastewater.

**Table 1:** The isolated marine-derived fungi

No	Code	Source
1	Lgd21d	Hurgada sea sediment
2	**Nve9**	Hurgada sea water site 1
3	Xld5	Hurgada sea water site 2
4	Mono35	Hurghada Abo monqar island sea water
5	Xld2	Hurgada sea water site 2
6	Cla	Sponge
7	Nv35	Hurgada sea water site 1
8	Hc2	Hurgada sea water site 3
9	Sl1a	Sponge
10	Nvef2	Hurgada sea water site 1

**Table 2:** The amount of bioethanol

	Area	Conc mg		Mg ml-1	ppm
Ethanol	64213045.6	39.45			
Waste	0.0		0.0		0.0
*P. chrysogenum Cla* with *S. cerevisiae Sc*	161669.52		0.10		**99.32**
*P. chrysogenum Mono35* and *S. cerevisiae Sc*	115449.04		0.07		70.93
*A.fumigatus Nv35* and *S.cerevisiae Sc*	96978.99		0.06		59.58

**Table 3:** Treatment efficiency of nutrient and organic load after *P.chrysogenum* spp. (Cla) and *S. cerevisiae* (Sc)treatment

Nutrients and organic load characteristics (mg l^− 1^)	Raw sample	Sample After *P. chrysogenum* spp (Cla) and *S. cerevisiae* (Sc) treatment	% Removal
NH_3_	320	320	0.0
NO_2_	47.2	47.2	0.0
NO_3_	60	60	0.0
PO_4_	136	50	63.2
COD	17,800	10,000	43.8
BOD	12,540	2400	80.8

**Table 4:** Treatment efficiency of nutrient and organic load after algal treatment

Nutrients and organic load characteristics (mg l^− 1^)	Diluted sample before algal treatment	Diluted sample after algal treatment	% Removal
NH_3_	186.75	14.025	92.5
NO_2_	0.065	0.06	7.7
NO_3_	78.3	4.63	94.1
PO_4_	27.69	0	100
COD	640	22	96.5
BOD	193	7.5	96.1

## Data Availability

No datasets were generated or analysed during the current study.
